# Individual Patterns of Complexity in Cystic Fibrosis Lung Microbiota, Including Predator Bacteria, over a 1-Year Period

**DOI:** 10.1128/mBio.00959-17

**Published:** 2017-09-26

**Authors:** Juan de Dios Caballero, Rafael Vida, Marta Cobo, Luis Máiz, Lucrecia Suárez, Javier Galeano, Fernando Baquero, Rafael Cantón, Rosa del Campo

**Affiliations:** a Servicio de Microbiología, Hospital Universitario Ramón y Cajal, and Instituto Ramón y Cajal de Investigación Sanitaria (IRYCIS), Madrid, Spain; b Red Española de Investigación en Patología Infecciosa (REIPI), Madrid, Spain; c Grupo de Sistemas Complejos, ETSIAAB, Universidad de Politécnica de Madrid, Madrid, Spain; d Unidad de Fibrosis Quística, Hospital Universitario Ramón y Cajal, Madrid, Spain; e CIBER en Epidemiología y Salud Pública (CIBERESP), Madrid, Spain; University of British Columbia

**Keywords:** antibiotic consumption, *Bdellovibrio*, cystic fibrosis lung microbiota, next-generation sequencing, predator bacteria, *Vampirovibrio*

## Abstract

Cystic fibrosis (CF) lung microbiota composition has recently been redefined by the application of next-generation sequencing (NGS) tools, identifying, among others, previously undescribed anaerobic and uncultivable bacteria. In the present study, we monitored the fluctuations of this ecosystem in 15 CF patients during a 1-year follow-up period, describing for the first time, as far as we know, the presence of predator bacteria in the CF lung microbiome. In addition, a new computational model was developed to ascertain the hypothetical ecological repercussions of a prey-predator interaction in CF lung microbial communities. Fifteen adult CF patients, stratified according to their pulmonary function into mild (*n* = 5), moderate (*n* = 9), and severe (*n* = 1) disease, were recruited at the CF unit of the Ramón y Cajal University Hospital (Madrid, Spain). Each patient contributed three or four induced sputum samples during a 1-year follow-up period. Lung microbiota composition was determined by both cultivation and NGS techniques and was compared with the patients’ clinical variables. Results revealed a particular microbiota composition for each patient that was maintained during the study period, although some fluctuations were detected without any clinical correlation. For the first time, *Bdellovibrio* and *Vampirovibrio* predator bacteria were shown in CF lung microbiota and reduced-genome bacterial parasites of the phylum *Parcubacteria* were also consistently detected. The newly designed computational model allows us to hypothesize that inoculation of predators into the pulmonary microbiome might contribute to the control of chronic colonization by CF pathogens in early colonization stages.

## IMPORTANCE

The application of NGS to sequential samples of CF patients demonstrated the complexity of the organisms present in the lung (156 species) and the constancy of basic individual colonization patterns, although some differences between samples from the same patient were observed, probably related to sampling bias. *Bdellovibrio* and *Vampirovibrio* predator bacteria were found for the first time by NGS as part of the CF lung microbiota, although their ecological significance needs to be clarified. The newly designed computational model allows us to hypothesize that inoculation of predators into the lung microbiome can eradicate CF pathogens in early stages of the process. Our data strongly suggest that lower respiratory microbiome fluctuations are not necessarily related to the patient’s clinical status.

## INTRODUCTION

The natural evolution of cystic fibrosis (CF) disease is a progressive decline in lung function caused by a vicious circle of inflammation and tissue destruction that is triggered and maintained by chronic bacterial colonization of the lower respiratory tract (1). The main pathogen detected in the CF airway by conventional culture techniques is *Pseudomonas aeruginosa*, which has a major influence on patients’ survival and quality of life (1, 2). Other clinically cultivable and relevant recognized pathogens include methicillin-resistant *Staphylococcus aureus* (MRSA), *Burkholderia cepacia* complex species, and *Mycobacterium abscessus*. The clinical impact of anaerobes, *Stenotrophomonas maltophilia*, *Achromobacter* species, *Ralstonia*, *Pandoraea*, *Acinetobacter*, and others remains unclear (2–4).

The introduction of culture-independent tools, particularly those based on next-generation sequencing (NGS), has allowed the identification of a more diverse and abundant lung microbiota that includes not only classical CF pathogens but also a wide community of bacterial taxa, most of which are uncultivable by routine methods (5–8). The precise role of this microbiota in the clinical course of the disease has not yet been elucidated, although its implication in the onset of clinical exacerbations and in the modulation of virulence factors of pathogens such as *P. aeruginosa* has been suggested (3, 9, 10). As in other mucosal compartments of the human body, the bacterial composition of the lung microbiota is patient specific. In CF, complexity and diversity reductions in the lung microbiota are often associated with progression of the disease. In addition, microbiota disturbances are mostly related to the cumulative effect of multiple courses of antibiotic treatment, although other factors, such as the host’s immune system, human metabolites, bacterial quorum-sensing molecules, the spread of bacteriophages among bacteria, and other ecological forces, are also implicated in these fluctuations (11). Bacteriophages are important negative bacterial density regulators and might also contribute to the survival of particular species through the horizontal transfer of genes, especially those related to antibiotic resistance (12).

In this study, we applied NGS to the study of the lung microbiota in successive sputum samples from 15 CF patients during a 1-year period, revealing a basic constancy in the pattern of species found in each patient. Unexpectedly, prokaryotic predators in the CF lung microbiota were found that might influence and/or regulate the composition of the microbiota and the population size of particular organisms. *Bdellovibrio* species are aerobic deltaproteobacteria, motile, very small (0.6 μm in diameter), Gram-negative rods, and obligate predators of both Gram-negative and Gram-positive bacteria (13, 14). After initial contact, the predator adheres to the bacterial envelope and enters the periplasmic space, where it forms its replicative structures, called bdelloplasts, by using the prey’s cytoplasm as an energy source (15). Once the cytoplasm is exhausted, the lysis of the remnant cell allows the exit into the medium of multiple *Bdellovibrio* cells that then search for other cells on which to prey (16). The most-studied predator species is *Bdellovibrio bacteriovorus*, which is widely distributed in environmental ecosystems and particularly linked to saltwater settings, in which an ecological role in the bacterial communities’ regulation has been suggested (15). The presence of *B. bacteriovorus* in the gut microbiota of healthy individuals and CF patients has been recently reported, as well as its ability to prey on classical CF pathogens such as *P. aeruginosa*, *S. aureus*, and *S. maltophilia*, even in their biofilm format (14, 16). For these reasons, the use of these predator bacteria as an ecological strategy to control pathogenic CF lung bacteria and also as a probiotic to control gut microbiota dysbiosis in inflammatory bowel disease has been suggested (17, 18). Interestingly, *S. maltophilia*, a frequent CF lung colonizer, has also been identified as a predator of the green sulfur bacterium *Chlorobium limicola* in aquatic environments (19). Moreover, *Vampirovibrio chlorellavorus* is an epibiotic bacterial predator whose known target is the microalga *Chlorella vulgaris* (20, 21). An unexpected finding of the application of NGS in the present study encouraged us to report for the first time, as far as we know, the presence of predator bacteria in the CF lung microbiome, monitoring their possible association with the population fluctuation in 15 CF patients during a 1-year follow-up period. In addition, a new computational model was developed to illustrate the possible ecological repercussions of prey-predator interactions in CF lung microbial communities.

## RESULTS

All 15 of the CF patients in this study completed the 1-year follow-up, contributing the four scheduled sputum samples, with the exception of four patients (no. 1, 10, 12, and 15), who contributed only three samples because of circumstances not related to this study. The patients were classified as having mild (*n* = 5), moderate (*n* = 9), or severe (*n* = 1) lung function impairment (Table 1).

**TABLE 1:** Clinical characteristics of the patients included in this study with the chronic and acute antibiotic treatments received during the study

Lung function impairment and patient no.	FEV_1_ (%)	Sex^a^	Age (yr)	Chronic treatment (route)^b^	Exacerbation treatment(s) (no. of incidents)			Cultured pathogens^c^
					Inhaled	Oral	Intravenous	
Mild								
1	90	F	40	TOB (inh), ATM (inh)		CIP (2), AZM (2)		Hp, Pa, Sm
2	87	M	39			MIN (2), MOX (2)	VAN, CFX	Sm, Hi, Sa, Hp
3	80	F	38	COL (inh)		CIP (6), FOS (3), SXT, AZM		Pa, Sw
4	80	M	19	COL (inh)		CFX (4), CIP		Sa, Hp, Hpitt, Pa
5	75	F	40	COL (inh)		CIP		Sa, Bv, Bc, Hp, Pa
Moderate								
6	73	M	36	COL (inh)	AMP	AMC (6), MOX (2)		Hp, Sm, Cp, Sl
7	62	F	32	COL (inh)		SXT (3), AMC	CIP, AMK	Sa, Ps, Hp, Hp, Mm
8	61	F	34	AZM (p.o.), ATM (inh)		AZM, FOS, LEV	TOB (2), PTZ, CFT	Hp, Pa, Sw, Sm
9	61	F	29	AZM (p.o.)	AMP	AMC (4)	MER, AMC	Sa, Hp, Hpitt, A
10	60	F	49		TOB	MOX (3), AMC, SXT (7), CLO (7), CIP		Sa, Sm, Ca, Pa, Af
11	56	M	39	AZM (p.o.), COL (inh)		SXT (2)		Sa, Pa, Hp
12	52	F	21	TOB (inh)		CIP (3), AMC (2), AZM		Sa, Pa
13	52	F	19	COL (inh)		AZM		Pa, Sa
14	46	F	22	TOB (inh), AZM (p.o.)		AMC (2), SXT, LNZ (2)	TOB, PTZ	Pa, Sa, Cg
Severe								
15	28	F	28	COL (inh)	Col, ATM	CIP (3), LEV, SXT, ATM	MER (3), TOB (4), FOS, PTZ (3)	Hp, Sa, Pa

a F, female; M, male.

b TOB, tobramycin; AZM, azithromycin; COL, colistin; CIP, ciprofloxacin; MIN, minocycline; MOX, moxifloxacin; LEV, levofloxacin; FOS, fosfomycin; SXT, trimethoprim-sulfamethoxazole; AMC, amoxicillin-clavulanate; ATM, aztreonam; CLO, cloxacillin; MER, meropenem; PTZ, piperacillin-tazobactam; CFX, cefuroxime; CTX, cefotaxime; LNZ, linezolid; VAN, vancomycin; inh, inhaled; p.o., *per os*.

c Sa, *Staphylococcus aureus*; Sw, *Staphylococcus warneri*; Sl, *Staphylococcus lugdunensis*; Pa, *Pseudomonas aeruginosa*; Hp, *Haemophilus parainfluenzae*; Hi, *Haemophilus influenzae*; Hpitt, *Haemophilus pittmaniae*; Sm, *Serratia marcescens*; Bv, *Burkholderia vietnamiensis*; Bc, *Burkholderia cepacia*; Ps, *Pandoraea sputorum*; Ca, *Candida albicans*; Cp, *Candida parapsilosis*; Cg, *Candida guilliermondii*; Mm, *Morganella morganii*; A, *Achromobacter*; Af, *Aspergillus fumigatus*.

### A wide range of bacterial species in the CF lung microbiota.

Microbiological cultures of the samples demonstrated chronic lung colonization by CF pathogens, including *P. aeruginosa* (11 patients), *S. aureus* (11 patients), *Burkholderia* species (1 patient), and *Pandoraea* species (1 patient). *P. aeruginosa* and *S. aureus* cocolonization was observed in the eight patients with the poorest lung function (Table 1). MRSA isolates were not detected.

**FIG 1:**
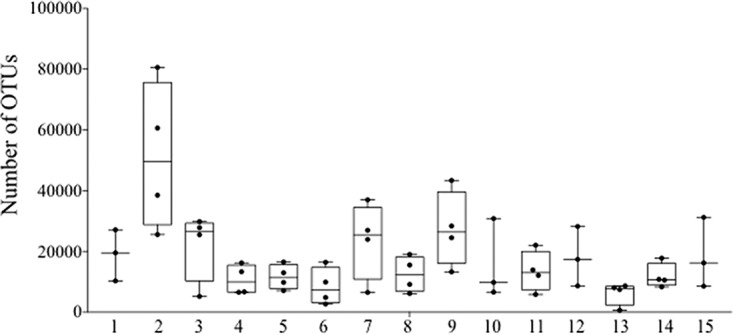
Biodiversity of the sputum samples used in this study. The median, minimum, and maximum numbers of OTUs in the samples are represented.

**FIG 2:**
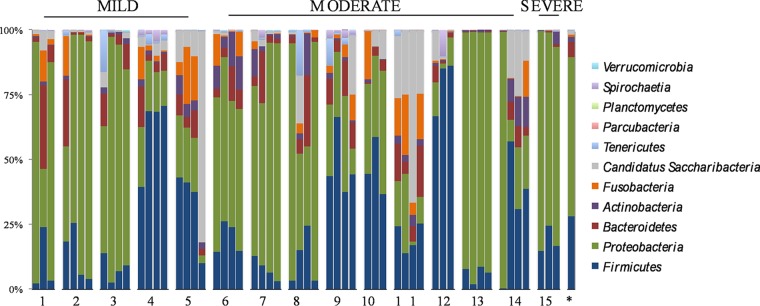
Phylum distribution in the samples from the 15 CF patients in this study. The last column (*) represents the median value of all 56 samples.

**FIG 3:**
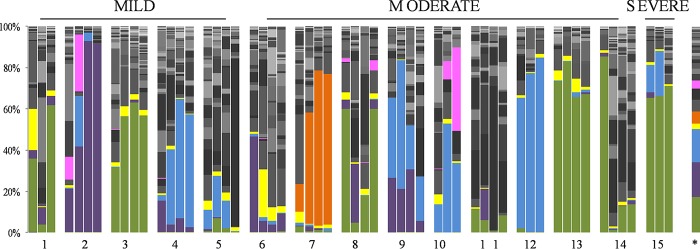
Genus percentages in the sequential sputum samples. Green, *Pseudomonas*; purple, *Haemophilus*; blue, *Staphylococcus*; yellow, *Burkholderia*; orange, *Pandoraeae*; and pink, *Stenotrophomonas*.

Oral-cavity-related microorganisms are usually considered contaminants after sputum passage through the upper respiratory tract and the mouth; thus, we decided to delete oral-cavity-related genera (*Actinomyces*, *Fusobacterium*, *Gemella*, *Granulicatella*, *Neisseria*, *Porphyromonas*, *Prevotella*, *Rothia*, *Streptococcus*, and *Veillonella*) from the final analysis to better monitor the lung microbiota (Fig. 1 to 3). The median number of operational taxonomic units (OTUs) in all of the samples was 16,780 ± 14,670 (range, 711 to 82,507); as expected, the lower number of reads corresponded to the patients with the poorest lung function (Fig. 1). Taxonomic genus assignment was incomplete in 10% of the total reads, and only the family taxon was ascribed to these sequences.

Fluctuations in phylum distribution are shown in Fig. 2, overall the most represented by *Proteobacteria* (62.1% ± 29%), *Firmicutes* (28.3% ± 21%), *Bacteroidetes* (5.7% ± 5%), *Actinobacteria* (1.9% ± 3%), and members of the ubiquitous phylum *Candidatus* Saccharibacteria (1.6% ± 12%). Other minority phyla (≤0.9%) were *Parcubacteria*, organisms of the new big bacterium phylum, present in all 15 patients, despite being able to grow only in anoxic environments; *Verrucomicrobia* and *Cyanobacteria/*chloroplasts in 13 patients; *Nitrospirae* in 10 patients; *Chlamydiae* in 9 patients; and *Armatimonadetes* in 8 patients. Although the phylum pattern was maintained in each patient over time, significant fluctuations were also observed, particularly in *Proteobacteria* in patients 1, 8, and 14, without any clinical association.

Considering all 56 samples, a total of 156 bacterial genera were detected, although ~90% of the CF lung microbiome consisted of *Pseudomonas* (18%), *Haemophilus* (17.3%), *Staphylococcus* (16.5%), *Pandoraea* (6.2%), *Sphingomonas* (5.8%), *Ca.* Saccharibacteria *genera incertae sedis* (5.5%), *Stenotrophomonas* (4.0%), *Leptotrichia* (3.2%), *Capnocytophaga* (3.0%), *Burkholderia* (2.4%), *Oribacterium* (2.1%), *Aquabacterium* (1.8%), *Lachnoanaerobaculum* (1.4%), *Campylobacter* (1.3%), and *Mycoplasma* (1.2%) (Fig. 3). Cultivable *Burkholderia* bacteria were observed only in the 4 sputum samples of patient 5, although compatible reads were detected by NGS in all 56 samples studied, with a median number of 436 ± 531 (range, 39 to 1,866) reads (Fig. 3). Unexpectedly, patient 5 displayed a low number of *Burkholderia* reads in the four samples (median of 368 ± 120 reads). Because the possible contamination of samples or reagents with ambient *Burkholderia* was prevented by using adequate negative controls, independent *gyrB* and *recA* PCRs were developed with the total DNA from the sputum, with negative results for all of the samples except those from patient 5.

### Predators in the CF lung microbiota.

**FIG 4:**
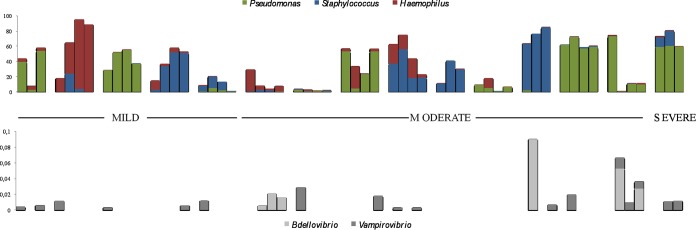
Numbers of OTUs of the main CF pathogens and predator species detected in the sputum samples.

Interestingly, NGS allowed the detection of some recognized bacterial predators as minority genera, i.e., *Vampirovibrio* (17 samples, 12 patients, 0.003% of the total microbiota) and *Bdellovibrio* (6 samples, 3 patients, 0.002% of the total microbiota). The detection of these predator organisms as obligate parasites implies the multiplication in the CF lung microbiota of prey; however, we were unable to find known prey organisms in the sputum samples that might explain the presence of *Vampirovibrio*. An association between the presence of predators and the main CF pathogen densities was not detected (Fig. 4). The coexistence of both predators in the same sputum sample was observed only in patient 14, with the most frequent situation the predator’s detection in a single sample from each patient.

### Modeling of predator-prey interactions in the CF lung microbiota.

**FIG 5:**
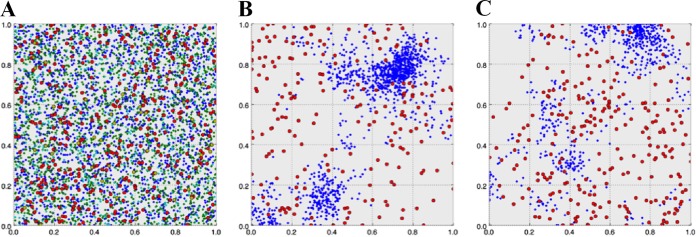
Spatial distribution of the bacteria in the computational model and evolution in time. (A) Random initial distribution of 5,000 bacteria of five different species (3,500 *Pseudomonas* [blue], 1,000 *Staphylococcus* [green], 350 *Haemophilus* [light blue], 100 *Bdellovibrio* [red], and 50 SPP [yellow] bacteria). (B) Spatial distribution at 2,500 arbitrary time units. (C) Spatial distribution at 5,000 arbitrary time units. Coexistence of *Pseudomonas* and *Bdellovibrio* was observed in evolved panels B and C after the other bacterial species disappeared.

A new computational model was designed to better understand the ecological interrelationships of predators and the CF lung microbiota. For this purpose, and considering the real proportions observed in our sputum samples, the bacteria selected as prey were *Pseudomonas* and *Staphylococcus*, whereas *Bdellovibrio* was the predator. Because of uncertainty about the role of *Vampirovibrio*, we decided to introduce a second putative predator (SPP). The spatial distribution of all of the agents in the three different stages of the temporal evolution is shown in Fig. 5, and the overall results obtained with this model at the arbitrary time points reproduce the classical oscillatory solution of the Lotka-Volterra equations and were consistent with the extinction of all populations except one predator and one prey, which ultimately coexist in equilibrium.

**FIG 6:**
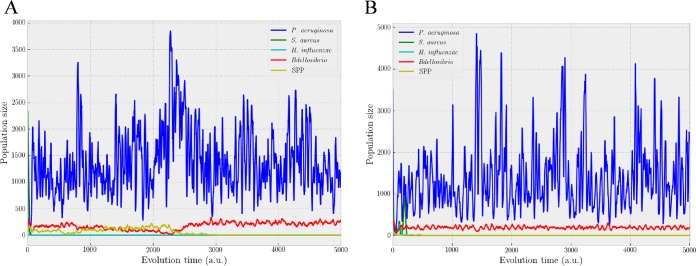
Temporal evolution by using arbitrary units of bacterial population size. (A) Initial distribution: 2,000 *Pseudomonas*, 2,000 *Staphylococcus*, 250 *Haemophilus*, 500 *Bdellovibrio*, and 250 SPP bacteria. (B) Initial distribution: 3,500 *Pseudomonas*, 1,000 *Staphylococcus*, 350 *Haemophilus*, 100 *Bdellovibrio*, and 50 SPP bacteria.

**FIG 7:**
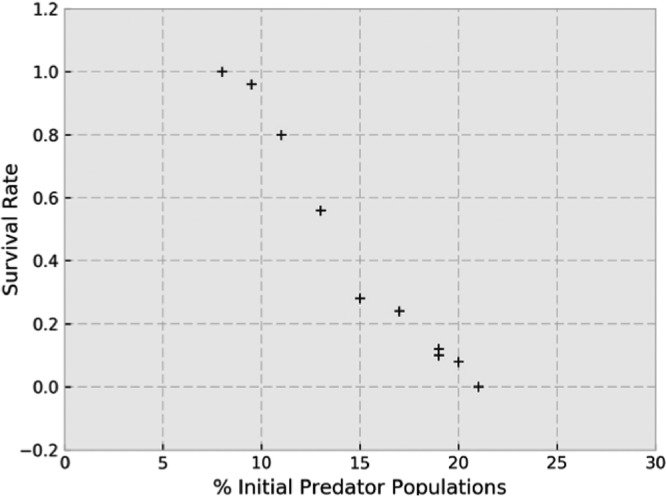
Survival rate versus initial percentage of predators to 50 repetitions. Initial populations that are <8% of the total bacteria in the simulations always survive, whereas initial populations that are >20% of the total bacteria always die.

Various combinations of the initial conditions were applied specially for the prey proportions (Fig. 6A and B), showing the ecological advantage of *Pseudomonas* with respect to *Staphylococcus*. To understand the influence of the initial populations of predators, we performed 50 repetitions of each simulation by studying whether populations survive or die by using the survival rate. Figure 7 shows that populations with an initial percentage of predators of <1% of the total bacteria in the simulations always survive, whereas populations with an initial predator percentage of >20% always die. A threshold appears in the simulations, and it becomes relevant if the objective is changing the final state of equilibrium, as is the case here.

## DISCUSSION

Chronic colonization of CF lungs by pathogenic bacteria has been extensively studied by using conventional microbiological cultures (1, 22), reporting the coexistence of several bacterial species at initial stages, as well as *P. areruginosa* dominance during the adult period (23, 24). Moreover, the lower bacterial diversity in the final stages of CF lung disease has been shown by both cultivation and molecular techniques (5, 25).

It is generally acknowledged that once bacterial colonization is established in the lung, its eradication is almost impossible, despite consistent antibiotic treatment. Nevertheless, the fluctuations observed in the bacterial populations from the early childhood period (dominated by *Haemophilus influenzae* and *S. aureus*) to the late childhood and adult periods (dominated by *P. aeruginosa*) must be explained by an ecological readjustment and their resilience properties (26–28). The association between lung colonization and the clinical status of the patient, particularly during exacerbations, has been explored without significant conclusions (6, 10, 29, 30).

Massive NGS strategies considerably increase the number of bacterial species that can be identified in respiratory samples (26, 28, 31), thus detecting the maintenance of a particular intraindividual pattern (32, 33). Although sputum is not the ideal sample, its easy and noninvasive sampling makes it the most universal sample for analysis of the lung microbiota. Sputum is always contaminated with upper respiratory microbiota (34, 35); however, continuous bronchial inoculation with oropharyngeal organisms also occurs, particularly in cases of wet cough. In addition, the lung is a highly compartmentalized space; therefore, the sputum microbiota is not necessarily homogeneous in composition.

An important factor, which is usually underestimated, is the use of a single respiratory sample per patient to decipher the lung microbiota. Because of the spatial heterogeneity of bacterial populations (32, 36), the representativeness of a single sample is controversial. We attempted to mitigate this by using four sputum samples per patient collected over a 1-year follow-up period, and although all of the patients appeared to maintain their own patterns, important variations in bacterial populations were observed without any apparent association with the clinical status of the patient or the antibiotic treatment.

A relevant finding is the detection of a *Burkholderia*-compatible OTU in all 15 patients, whereas cultivable isolates were detected in only 1. A possible explanation for this phenomenon is that the nucleotide sequence of the complete 16S rRNA gene can be insufficient to reach an adequate *Burkholderia* species identification and must be complemented with the multilocus sequence typing (MLST) *recA* and *gyrB* alleles (37), as performed in our study. These confusing results are also linked to the NGS platform, as demonstrated by Hahn et al., in whose study *Burkholderia* was detected only by PacBio RSII (full 16S rRNA gene) and not by MiSeq (V4 region) (38). Others have also reported *Burkholderia*-compatible reads in NGS of sputum samples (31, 39). Demonstration of lung colonization by this genus has a relevant clinical value that leads to instauration of isolation measures and antibiotic therapy. At present, only conventional culture methods guarantee the presence of a significant number of cells; intragenic regions of the 16S rRNA gene have poor specificity for *Burkholderia* species identification (38).

The presence of members of the phylum *Parcubacteria* in CF lungs has not yet been highlighted. These reduced-genome bacterial organisms comprise more than 15% of the entire *Bacteria* domain (40, 41) and might be maintained as “small-genome” parasites of other bacteria. Interestingly, they share with predator bacteria such as *Bdellovibrio* the type IV pilus operon involved in cell-to-cell contact and predation (42, 43).

The most unexpected result of the present study was the consistent detection of the classic predator bacteria *Bdellovibrio* and *Vampirovibrio* as part of the CF lung microbiota. The natural lifestyle of these predators is related to aquatic environments, because they prey on Gram-negative bacteria and *Chlorella*, respectively. We hypothesize that lung colonization by both prey and predator bacteria might occur in a single event from their natural reservoir. *B. bacteriovorus* has also been described as a frequent component of the gut microbiome of healthy individuals and CF patients (16). These findings suggest that *B. bacteriovorus* could ultimately be involved in the biotic regulation of the human gut microbiota, and its possible usefulness as a probiotic has been suggested (18, 44). The presence of other predators, such as *Micavibrio aeruginosavorus* (45), in other CF patients cannot be ruled out.

To our knowledge, this is the first time that *Bdellovibrio* and *Vampirovibrio* are being reported as part of the CF lung microbiota. The reason is possibly the fact that the predator population is much less abundant than that of potential prey, making its detection difficult by NGS techniques that fail to discover low-density taxa. The presence of *Bdellovibrio* in CF lungs could be explained by acquisition from the environment, although other possibilities cannot be ruled out, including aspiration of gastrointestinal content. These bacteria appear to be particularly abundant in the duodenum, as shown in the above-mentioned gut microbiome study (16); also, there is a gut-lung axis, which justifies the continuous connection between the two ecosystems (46, 47). CF lungs should, in theory, be a good habitat for *B. bacteriovorus*, which needs a minimal prey density of 10^5^ to 10^6^ CFU/ml (48). In addition, this predator has been shown to be able to survive the anoxic conditions found in the CF lung and during the bdelloplast phase is protected against the phage’s attack and possibly the effects of antibiotics. Its ability to feed on typical CF pathogens such as *P. aeruginosa* and *S. aureus* has been reported (14), including when the pathogens are growing in biofilms (49).

Scientific data on the second predator found, *Vampirovibrio*, which also is a *Cyanobacteria*, remain scarce; *V. chlorellavorus* is the only reported species (20, 50). This is an anaerobic, nonphotosynthetic cyanobacterium that needs the presence of microalgae to grow, particularly the eukaryotic species *Chlorella vulgaris*. On the basis of our current knowledge, the presence of *Vampirovibrio* in the CF lung implies the presence of *Chlorella*. Because *Chlorella* is frequent in the air, particularly in freshwater environments (51, 52), it can easily be introduced into the CF lung by the extremely frequent aerosol inhalation therapies (53) or assisted ventilation used by these patients. The existence of algae in the CF lung has not yet been explored; however, we can speculate on the possibility that the dense microbiota of the lung in CF patients might produce mutually beneficial interactions with *Chlorella*, as has been described for other bacterial species (54). *Chlorella*-bacterial biocenosis has been reported (55, 56), particularly with *Pseudomonas*, and curiously, the alga-bacterium mutual growth promotion appears to occur on immobilized alginate surfaces (57). These interactions might eventually result in the replication of *Chlorella* in the CF lung and permanent colonization, explaining the presence of *Chlorella* predators such as *Vampirovibrio*; however, we were unexpectedly unable to detect *Chlorella* in our samples. At the moment, we cannot exclude the presence of unknown *Vampirovibrio* prey in the lung microbiota. In addition, the unexpected detection of *Cyanobacteria* by NGS techniques in an intensive care unit environment has been reported (58), suggesting that algae can be part of the habitual human microbiome. In fact, 13 of our 15 CF patients carried *Cyanobacteria* in their lung microbiome.

Using the computational model, the persistence and coexistence of a prey-predator couple was observed over time with ecological alternation of both prey species, probably reproducing the real ecological CF lung battle. The most promising results obtained with the computational model are related to a high initial proportion of the predator, simulating an artificial addition as could happen if predators were used as a biological weapon. All of the bacterial species were eliminated with this condition; first, prey are destroyed by predators and then predators become extinct because of the absence of nutritional sources, suggesting that *Bdellovibrio* might eventually be used as a “biological antibiosis strategy” to control pathogenic bacterial populations (59), at least in the early stages of the colonization process, when the prey density is still low. The safety of these microorganisms has been demonstrated in animals (60), in human cells lines (61, 62), and in rat lungs (63), in which reversible weak inflammation is the only adverse effect observed. On the other hand, as occurs with bacteriophages, prey could develop a natural resistance to the predator. This resistance has been described during *in vitro* experiments, although it appears to be transient, with the bacterium recovering its original susceptibility in a short period of time without exposure to predators (64).

In summary, we show the complexity of the organisms present in the CF lung (156 species) and the constancy of basic individual colonization patterns. *Bdellovibrio* and *Vampirovibrio* predator bacteria were found for the first time by NGS as part of the CF lung microbiota, although their ecological significance needs to be clarified. The newly designed computational model allows us to hypothesize that the inoculation of predators into the lung microbiome can eradicate CF pathogens in the early stages of the process. Our data strongly suggest that lower respiratory microbiome fluctuations are not necessarily related to the patient’s clinical status.

## MATERIALS AND METHODS

### Patients.

Fifteen adult CF patients regularly attending our CF unit were recruited, and each contributed three or four induced sputum samples during a 1-year follow-up period. Immediately after collection, the samples were separated into two aliquots, one for conventional culture processing and the other for 16S rRNA gene NGS, and frozen at −80°C. Clinically relevant data on the patients included in this study are shown in Table 1. Patient selection included no restrictive criteria, except a solid commitment to the study. The patients were stratified according to their pulmonary function, measured by the percentage of predicted forced expiratory volume in 1 s (FEV_1_) as follows: advanced disease, <40%; moderate disease, 40 to 70%; mild disease, 70 to 90%; normal lung function, >90%. Our hospital’s ethics committee approved this study, and all of the participants provided written informed consent.

### Microbiological culture.

Sputum samples were routinely aerobically cultured in accordance with the recommendations of the Spanish CF guidelines (65). Briefly, after homogenization with *N*-acetyl-l-cysteine and/or sterile saline solution, the samples were qualitatively and quantitatively used to seed (10^−2^ and 10^−4^ dilutions) general media (Columbia agar with sheep blood and with chocolate horse blood) and selective/differential media (MacConkey agar, *B. cepacia* selective agar, mannitol-salt agar, bacitracin chocolate agar, and Sabouraud chloramphenicol agar). Incubation times were prolonged from 48 h at 37°C to 5 days at room temperature for bacteria and 30 days at 30°C for fungi. The chocolate agar was incubated in a 5% CO_2_ atmosphere. All colonies were identified by matrix-assisted laser desorption ionization time of flight mass spectrometry (Bruker Daltonik, Germany), except those of the *B. cepacia* complex, whose identification to the species level was based on a *recA* and *gyrB* MLST profile (37). The identification of nonfermenting Gram-negative rod species, including *Ralstonia* species, *Cupriavidus* species, *Elizabethkingia* species, *Rahnella* species, and *Pandoraea* species, was confirmed by Sanger 16S rRNA gene sequencing. Filamentous fungi were identified microscopically by lactophenol cotton blue staining.

### 16S rRNA gene NGS.

The sputum samples were slowly defrosted at 4°C for 24 h to prevent DNA degradation and further thawed at room temperature. After complete vortex mixing of the sample, total DNA was obtained from an aliquot of ~0.5 ml of the supernatant with the QIAamp DNA minikit (Qiagen, Germany). DNA samples were sent to FISABIO (Valencia, Spain) for massive 16S rRNA gene V3-V4 amplicon sequencing on the Illumina MiSeq platform and for bioinformatic analysis. The Shannon index was used for estimation of bacterial diversity. Taxonomic affiliations were assigned by using the Ribosomal Database Project (RDP) classifier, and reads with an RDP score of <0.8 were assigned to the upper taxonomic rank, leaving the last rank unidentified. The statistical analysis was performed with R statistical software and several open-source libraries. The quantitative data of the reads were homogenized by using their relative percentages of the total reads of each sample to facilitate the comparison between samples.

### Computational predator-prey model.

A new multiple computational model based on the traditional Lotka-Volterra equations (66, 67) was designed to predict the ecological significance of prey-predator interactions over time with the free software available at https://github.com/galeanojav/LV_5species. The features considered in the model included that the bacteria be discretely defined and spatially distributed and could duplicate and die during the simulation. Enough arbitrary time points were defined (5,000), and it is important to note that at each time point we had to update each of the agents, at least on average. On the basis of the real bacterial proportions observed in the sputum samples from our CF patients, we introduced into our simulations a total of 5,000 “bacterial cells” with two defined roles, prey (*Pseudomonas* and *Staphylococcus*), ranging from 80 to 99% of the population, and predators (*Bdellovibrio* and an SPP), ranging from 1 to 20%. All of the agents were randomly dispersed spatially, and their reproduction rates were fixed. The main rules to define the agents’ behavior and their interactions were as follows. (i) If a prey cell met a predator cell, the prey cell died. (ii) On the other hand, if the predator could not find any prey spatially close, the predator died with some probability. (iii) If the predator could feed on prey, it resulted in the predator’s reproduction at a certain growth rate. (iv) Both types of agents could diffuse randomly in the space.
